# Undiagnosed Delusional Infestation Leading to Severe Self-Inflicted Injuries: A Case Report

**DOI:** 10.1155/crps/8626785

**Published:** 2025-11-21

**Authors:** Xiaofeng Yan, Kirsi Anselmi-Stith, James A. Bourgeois

**Affiliations:** ^1^Department of Dermatology, University of California, Davis Medical Center, Sacramento, California, USA; ^2^Department of Psychiatry and Behavioral Sciences, University of California, Davis Medical Center, Sacramento, California, USA

## Abstract

**Background:**

Delusional infestation (DI), or delusional disorder, somatic type, is a rare psychiatric condition characterized by a fixed false belief of infestation despite lack of medical evidence. Patients frequently resist psychiatric care due to poor insight and somatic preoccupation, increasing the risk of delayed diagnosis and serious self-harm.

**Case Presentation:**

We report the case of a 60-year-old woman with no prior psychiatric history who presented with 11.5% total body surface area (TBSA) second- and third-degree self-inflicted burns resulting from attempts to eradicate perceived skin parasites. She reported a 2-year history of pruritus attributed to “shiny fiberglass bacteria,” with associated tactile hallucinations. Extensive medical workup excluded underlying anatomic, inflammatory, or infectious etiologies. The psychiatry consultant diagnosed her with delusional disorder, somatic type. Treatment with low-dose risperidone and doxepin was initiated following empathic, nonconfrontational engagement. Her distress improved, and she demonstrated early signs of insight by the time of hospital discharge.

**Conclusion:**

This case highlights the risk of severe self-injury in DI and underscores the need for timely psychiatric evaluation and a compassionate, collaborative approach. Reframing treatment goals to prioritize symptom relief over delusional challenge may enhance engagement and facilitate recovery.

## 1. Introduction

Delusional infestation (DI), previously known as delusional parasitosis, is classified as delusional disorder, somatic type in the DSM-5-TR [1] and as a subtype of delusional disorder in the ICD-11. It is characterized by a fixed and false belief of infestation with parasites, insects, or other organisms despite the absence of medical evidence [2]. DI may be primary or secondary to medical or substance-induced causes. Patients commonly present with excoriations, ulcerations, or burns due to excessive scratching, picking, or chemical use in attempts to “remove” the infestation. Tactile hallucinations described as “crawling, biting, or stinging” often accompany these beliefs [3]. Despite the clear need for psychiatric care, patients frequently resist psychiatric consultations or referrals due to poor insight and fixed somatic beliefs. Empathic, nonconfrontational engagement is essential to build trust and facilitate effective treatment. This case highlights a severe presentation of previously undiagnosed DI, resulting in life-threatening self-inflicted injuries requiring surgical intervention.

## 2. Case Presentation

Ms. M. was a 60-year-old woman with no prior psychiatric history and a medical history of type 2 diabetes mellitus, neuropathy, and rheumatoid arthritis. She presented to the emergency department with extensive wounds on her back and buttocks. Physical examination and burn surgery consultation evaluation revealed 11.5% total body surface area (TBSA) mixed second- and third-degree burns. The patient stated these wounds had developed insidiously over 2 years and had not responded to multiple courses of oral and topical antibiotics and antifungals prescribed by her primary care provider and dermatologists. She was admitted to the burn surgery intensive care unit for wound management and surgical intervention. Psychiatry was consulted for suspected self-inflicted injury secondary to delusions.

**Figure 1:**
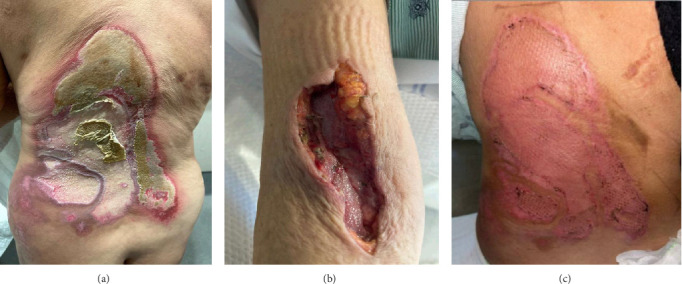
Self-inflicted wounds on the patient's back and right arm. (A) Chemical burn injury (2 days preadmission): patient applied bleach to her back in an attempt to “melt away” perceived bacteria. (B) Razor-induced skin trauma (1 week preadmission): patient shaved the surface of her skin with a razor to remove the perceived infestation. (C) Post-surgical site: status post tangential excision with split-thickness skin grafting on the back.

During the psychiatric interview, Ms. M. described an intense itch beginning 2 years prior, which she attributed to spider bites, her cat, or a resistant infection. She claimed to see “shiny fiberglass bacteria” on her skin after wiping with cotton balls. Days before admission, she applied bleach to “melt away the bacteria” (Figure 1A) and shaved the top layer of her skin with a razor blade (Figure 1B). She initially expressed pride in these actions, viewing them as necessary for self-preservation.

Mental status examination revealed a dysphoric mood, irritable affect, linear thought process, and fixed somatic delusions. She reported tactile hallucinations of “crawling bacteria,” but denied auditory or visual hallucinations. cognition was grossly intact.

Extensive medical and toxicological workup ruled out infectious, metabolic, neurologic, or substance-induced causes. She was diagnosed with delusional disorder, somatic type, first episode, currently in acute episode, severe. The psychiatric team employed a nonjudgmental and empathic approach, emphasizing validation of distress rather than direct confrontation of delusional beliefs. Daily rapport-building proved to be critical. Initially resistant to psychotropic treatment, Ms. M. ultimately agreed to try medications after they were framed as decreasing her distress about “bugs and infections” rather than changing her perceptions, per se. She was started on doxepin 10 mg at bedtime (QHS) for pruritus and risperidone 0.5 mg QHS for delusional distress, later titrated to 2 mg QHS.

By the time of discharge following burn surgery (Figure 1C), she expressed regret over having used bleach—a sign of emerging insight—and reported reduced distress. She was receptive to psychoeducation regarding long-acting injectable (LAI) antipsychotics and was provided with written materials outlining risks, benefits, and administration. However, LAI initiation was deferred due to the brief hospitalization and delayed discharge planning. Outpatient psychiatric follow-up was highly recommended, and Ms. M. voiced a willingness to engage in ongoing care.

## 3. Discussion

DI most commonly affects middle-aged to older women, with an approximate female-to-male ratio of 3:1 and an average age of onset around 57. Though cognitive function is often preserved, studies indicate that a substantial proportion of patients with DI have comorbid psychiatric conditions, including depressive, anxiety, and substance use disorders [4]. The annual incidence of DI is estimated to range from 2.37 to 17 cases per million, with a higher prevalence noted among older adults. This increased prevalence may be related to age-associated factors, such as social isolation, sensory decline, or neurobiological vulnerabilities [2, 4–6].

This case illustrates how untreated or unrecognized DI can escalate to life-threatening self-injury. Early recognition and multidisciplinary collaboration—particularly among dermatology, psychiatry, and primary care—are essential for optimal outcomes [7].

Validating the patient's distress without directly confronting the delusional content—and reframing treatment goals in patient-centered language—can significantly enhance engagement, adherence, and therapeutic alliance. Patients with fixed false beliefs, such as those with DI or other somatic delusions, often suffer from intense emotional distress and functional impairment. Directly challenging the delusion itself may result in mistrust, defensiveness, or refusal of care. Instead, clinicians should focus on acknowledging the patient's suffering (e.g., expressing understanding of how distressing or exhausting their experience has been) without affirming the false belief. This approach allows patients to feel heard and respected, which reduces resistance and fosters rapport [8, 9].

Reframing treatment in terms that align with the patient's subjective experience (e.g., “Let's focus on decreasing the discomfort and improving your sleep and functioning”) offers a collaborative pathway into treatment, without requiring the patient to abandon their delusional belief prematurely. This method respects the patient's autonomy and opens the door for the introduction of evidence-based therapies.

Psychotropic medications—particularly second-generation antipsychotics such as risperidone [10]—serve a dual therapeutic role in the treatment of DI. They can reduce the intensity and frequency of distressing somatic sensations (e.g., tactile hallucinations and abnormal skin sensations), while also gradually weakening the intensity of the delusional belief. A recent analysis by Tang et al. [11] identified amisulpride as the most effective treatment for DI; however, it is not currently available in the United States. Risperidone, as used in this case, ranked second in efficacy. LAI antipsychotic formulations may further support treatment by enhancing adherence, particularly in patients with limited insight or inconsistent follow-up. While cognitive-behavioral therapy (CBT) has been proposed as an adjunctive strategy to improve distress tolerance and coping, the evidence for its efficacy in DI remains limited and inconclusive.

## 4. Conclusion

Ms. M.'s case underscores the potential severity and clinical complexity of DI and its potential to result in devastating self-inflicted injury if left unrecognized or untreated. A comprehensive and empathic approach—grounded in validation, collaborative goal-setting, and well-framed and targeted pharmacologic intervention—can create a therapeutic environment where patients are more likely to engage in care, even when their insight into the nature of their symptoms remains limited. Early, prompt psychiatric involvement is vital to prevent escalation, mitigate the risk of serious self-inflicted harm, and promote long-term recovery.

## Data Availability

The data that support the findings of this study are available on request from the corresponding author. The data are not publicly available due to privacy or ethical restrictions.
